# Cesarean Section Is Associated with Increased Peripheral and Central Adiposity in Young Adulthood: Cohort Study

**DOI:** 10.1371/journal.pone.0066827

**Published:** 2013-06-27

**Authors:** Denise N. Mesquita, Marco A. Barbieri, Helena A. S. Goldani, Viviane C. Cardoso, Marcelo Z. Goldani, Gilberto Kac, Antônio A. M. Silva, Heloisa Bettiol

**Affiliations:** 1 Department of Puericulture and Pediatrics, Faculty of Medicine of Ribeirão Preto, University of São Paulo, Ribeirão Preto, Brazil; 2 Department of Pediatrics and Puericulture, Faculty of Medicine, Federal University of Rio Grande do Sul, Porto Alegre, Brazil; 3 Department of Social and Applied Nutrition, Federal University of Rio de Janeiro, Rio de Janeiro, Brazil; 4 Department of Public Health, Federal University of Maranhão, São Luís, Brazil; CUNY, United States of America

## Abstract

**Background:**

Cesarean section (CS) has been associated with obesity, measured by body mass index (BMI), in some studies. It has been hypothesized that this association, if causal, might be explained by changes in gut microbiota. However, little is known about whether CS is also associated with increased adiposity as measured by indicators other than BMI.

Objective: To assess the association between CS and indicators of peripheral and central adiposity in young adults.

**Methods:**

The study was conducted on 2,063 young adults aged 23 to 25 years from the 1978/79Ribeirão Preto birth cohort, São Paulo, Brazil. CS was the independent variable. The anthropometric indicators of adiposity were: waist circumference (WC), waist-height ratio (WHtR), waist-hip ratio (WHR), tricipital skinfold (TSF), and subscapular skinfold (SSF). The association between CS and indicators of adiposity was investigated using a Poisson model, with robust adjustment of variance and calculation of incidence rate ratio (IRR) with 95% confidence interval (95%CI), and adjustment for birth variables.

**Results:**

Follow-up rate was 31.8%. The CS rate was 32%. Prevalences of increased WC, WHtR, WHR were 32.1%, 33.0% and 15.2%, respectively. After adjustment for birth variables, CS was associated with increased risk of adiposity when compared to vaginal delivery: 1.22 (95%CI 1.07; 1.39) for WC, 1.25 (95%CI 1.10;1.42) for WHtR, 1.45 (95%CI 1.18;1.79) for WHR, 1.36 (95%CI 1.04;1.78) for TSF, and 1.43 (95%CI 1.08;1.91) for SSF.

**Conclusion:**

Subjects born by CS had a higher risk for increased peripheral and central adiposity during young adult age compared to those born by vaginal delivery. The association of CS with adiposity was consistently observed for all indicators and was robust after adjustment for a variety of early life confounders.

## Introduction

Over the last years, the prevalence of overweight and obesity has increased in the world population [1], [2]. A systematic analysis of the worldwide trends in body mass index (BMI) for adults 20 years old and older in 199 countries and territories between 1980 and 2008 showed that, despite a substantial variation in BMI between nations, mean BMI has increased on average 0.4 kg/m2 per decade for men and 0.5 kg/m2 per decade for women over the period [3].

This increase is also observed in the excessive accumulation of subcutaneous and visceral fat, which greatly contributes to metabolic complications and to adverse effects on health [4]. The accumulation of visceral or central fat has been shown to be a better predictor of adult morbidity than obesity alone measured by BMI, justifying the use of indicators related to visceral fats such as waist circumference (WC) and waist/height ratio (WHtR) [5].

Recent studies have shown that cesarean section (CS) is associated with a greater BMI both in children and adults [6], [7], [8], [9], [10]. A recent systematic review and meta-analysis including two case-control and seven cohort studies indicates that CS represents 33% higher risk of overweight and obesity for the offspring and 50% for adults 19 years old or older when compared to vaginal deliveries [11]. Gut microbiota seems to be an important factor connecting genes, environment, and the immune system [12]. Type of delivery seems to play a role in the composition of the intestinal microbiota in early infancy and this may be an environmental factor that modulates obesity and other metabolic diseases [13]. The mechanism by which CS may contribute to a greater risk of obesity appears to be based on changes in gut microbiota due to lack of contact of the baby with the maternal vaginal flora [14]. During vaginal delivery the baby is exposed to a wide variety of microorganisms, a fact that does not occur during CS [15], [16]. It has been hypothesized that this may lead to obesity in later life, probably due to increased absorption of fat and possibly by induction of low-grade inflammation [17], [18], [19].

To our knowledge, only one study [10], conducted on children, used measurements of adiposity other than the BMI, i.e., tricipital and subscapular skinfolds, to explore the association between CS and adiposity in early life. It was observed that babies born by CS had a 0.94 mm (95% CI 0.36 to 1.51) increment in the sum of skinfolds; however, CS was not associated with the subscapular/triceps skinfold ratio (β −0.18, 95% CI −2.30 to 1.94), a measure of central adiposity, when compared to those delivered vaginally.

A previous study on a cohort of young Brazilian adults showed an association between CS and total obesity measured by BMI [7]. To date, little is known about whether CS is also associated with increased central adiposity. Thus, the objective of the present study was to investigate in this same cohort whether babies born by CS have a higher risk for increased peripheral and central adiposity measured on the basis of indicators other than BMI.

## Methods

This was a prospective cohort study including live-born neonates in the city of Ribeirão Preto/São Paulo, from June 1978 to May 1979 [20]. During this period, 9,067 live neonates born in the eight Ribeirão Preto hospitals (98% of the total number of live newborns during the period) participated in the study. There were 3.5% losses due to refusal or early discharge from hospital. Babies whose mothers did not reside in the city and were not from Ribeirão Preto at the time of delivery were excluded, with 6,973 live newborns remaining, 6,827 of them singletons and 146 twin deliveries.

The cohort was re-evaluated between April 2002 and May 2004 when the individuals had completed 23–25 years of age. Of these, 246 died during the first year of life [21] and 97 died by 20 years of age, for a total of 343 deaths [22], leaving 6,484 eligible subjects. Contact was sought with one in each three individuals based on the geo-economic characterization of the city, divided into four regions, according to family head income. Based on the records of the Unified Health System and of private health plans and on the contacts made in the 2nd and 3rd phase of the study, it was possible to locate 5,665 individuals. The losses due to refusal to participate in the study (209 cases), to death after 20 years of age (34 cases), imprisonment (31 cases) and failure to attend the interview (431 cases) corresponded to a total of 705 individuals. Losses were replaced using the same sampling frame, resulting in 2,063 young adults aged 23 to 25 years, corresponding to 31.8% of the 6,484 subjects, participating in the 4th phase of the study of the Ribeirão Preto cohort [23].

The final sample consisted of 2,063 participants. This sample size permitted us to detect a 6% difference in the increased prevalence of adiposity between CS and vaginal delivery, assuming a prevalence of about 30%, with an 80% power and a 5% probability of type I error. For prevalence around 10% this same sample size permits the detection of 4% differences with the same power and the same probability of type I error. Details of the methodology have been previously published [23], [24].

The mothers were interviewed soon after delivery using a questionnaire with socioeconomic and demographic information. The newborns were weighed by trained personnel, using standardized techniques [25]. Gestational age was calculated on the basis of the mother's information about the last normal menstrual period.

The young adults were interviewed in order to obtain socioeconomic, demographic and life habit information. The following anthropometric measurements were obtained: weight, height, waist and hip circumference, and tricipital and subscapular skinfolds using standardized techniques applied by trained personnel. All measurements were obtained with the subjects wearing light clothing and no shoes.

WC was measured at the midpoint between the last rib and the upper margin of the iliac crest using an inextensible metric tape [26] and classified as increased when its value was ≥90 cm for men and ≥80 cm for women, as proposed by the International Diabetes Federation [27].

Height was measured with the individual standing up and barefoot, using a wood stadiometer with a wood support and an inextensible ruler. The subject stood up erect, with arms along the body and head on the Frankfurt plane [25].

WHtR was calculated as waist circumference in cm divided by height in cm and was defined as increased for men and women when its value was >0.5 [28].

Hip circumference was measured at the point of greater circumference on the gluteal region using an inextensible tape [25]. The waist-hip ratio (WHR) was calculated by dividing waist circumference in cm by the hip circumference in cm, and was considered to be increased when its value was ≥0.90 for men and ≥0.85 for women [29].

The tricipital skinfold (TSF) was measured in the posterior midpoint of the arm between the acromion and olecranon and the subscapular skinfold (SSF) was measured 2 cm below the margin of the lower angle of the scapula [25] using a caliper (Holtain Ltd., Crynych, U.K.,) with a limit measurement of 40 mm. Values above the 90th percentile obtained for the study population were considered to be increased.

The birth variables selected were birth weight (<2500 g, 2500 |–3000 g, 3000 |–3500 g, 3500 |–4000 g and ≥4000 g), type of delivery (vaginal and cesarean), newborn's sex, maternal schooling in years of study (0–4, 5–8, 9–11 and ≥12), maternal smoking during pregnancy as number of cigarettes smoked per day (non-smoker, 1–10, >10), parity (1, 2–4, ≥5), maternal age (<20, 20–34 and ≥35 years) and gestational age as a continuous variable.

The association of type of delivery with increased WC, WHtR, WHR, TSF and SSF was estimated by Poisson regression with robust adjustment of variance, with the calculation of the incidence rate ratio (IRR) and its respective 95% confidence interval (95% CI) [30], [31], and with the level of significance set at 0.05. The independent variables listed above were first submitted to non-adjusted analysis for each response variable; next, adjusted analyses were carried out, with the type of delivery being the explanatory variable and the remaining variables being possible confounders. Since there was selective attrition according to some birth variables, probabilities of selection for each individual were calculated in a logistic regression model. In this model those followed-up were coded 1 and those not followed up were coded 0. Maternal schooling, sex, maternal smoking during pregnancy and parity were predictors of the probability of participation in the follow-up. To verify if these different probabilities of selection would have biased the estimates, models using inverse-probability weighting were then fitted and compared with estimates derived from models without weighting [32].

Four models were fitted for each response variable. The first was the unadjusted model. The second was the unadjusted model using inverse-probability weighting. The third model was adjusted for birth variables (newborn's weight and sex, maternal schooling, maternal smoking during pregnancy, parity, maternal age and gestational age), and the last model was adjusted for birth variables using inverse-probability weighting. No significant interactions were detected between sex and the remaining adjustment variables. All analyses were carried out using Stata, version 12. Model fit was evaluated by the goodness of fit chi-squared test.

The study was approved by the Research Ethics Committee of the University Hospital, Faculty of Medicine of Ribeirão Preto, University of São Paulo (protocol HCRP n. 7606/99). All subjects gave written informed consent to participate in the study.

## Results

There was selective attrition. Males and individuals whose mothers were smokers, had ≥5 deliveries or had low schooling at the time of their birth were less likely to be interviewed (Table 1).

**Table 1:** Comparison of birth characteristics of those followed-up with those not followed-up in early adulthood.

Variables	Not followed-up (n = 4,421)*		Followed-up (n = 2,063)*		P-value**
	n	%	n	%	
Type of delivery					0.055
Vaginal	3,108	68.9	1,402	31.1	
Cesarean	1,312	66.5	661	33.5	
Maternal schooling (years)					<0.001
≥12	440	67.2	215	32.8	
9–11	542	62.1	331	37.9	
5–8	1,053	65.4	557	34.6	
0–4	2,266	71.1	920	28.9	
Sex					0.004
Female	2,117	66.5	1068	33.5	
Male	2,304	69.8	995	30.2	
Birth weight (grams)					0.618
<2500	252	66.3	128	33.7	
2500 |–3000	935	69.3	414	30.7	
3000 |–3500	1,796	67.9	848	32.1	
3500 |–4000	1,149	68.7	524	31.3	
≥4000	289	66.0	149	34.0	
Maternal smoking during pregnancy (cigarettes/day)					<0.001
Non-smoker	3,004	66.6	1509	33.4	
1–10	730	69.7	318	30.3	
>10	538	75.7	173	24.3	
Parity					<0.001
1	1,525	66.4	771	33.6	
2–4	2,247	67.5	1083	32.5	
≥5	511	75.2	169	24.8	
Maternal age (years)					0.065
<20	635	71.4	254	28.6	
20–34	3,372	67.5	1626	32.5	
≥35	366	68.2	171	31.8	

* Totals may not add up to 6,484 because of missing values.

** P-value refers to the chi-squared test.

1978/89 Ribeirão Preto birth cohort, 2002/2004.

The prevalence of increased WC, WHtR, WHR were 32.1%, 33.0% and 15.2%, respectively for the 2,063 young adults evaluated (Table 2).

**Table 2:** Distribution of the indicators of increased adiposity of young adults

Anthropometric indicators	N	%
**Waist circumference (WC)**		
Increased*	662	32.1
Not increased	1,399	67.8
Not known	2	0.1
**Waist-height ratio (WHtR)**		
Increased**	681	33.0
Not increased	1,375	66.6
Not knoun	7	0.4
**Waist-hip ratio (WHR)**		
Increased***	314	15.2
Not increased	1,746	84.6
Not knoun	3	0.2
**Tricipital skinfold (TSF)**		
Increased****	207	10.0
Not increased	1,855	89.9
Not knoun	1	0.1
**Subscapular skinfold (SSF)**		
Increased****	194	9.4
Not increased	1,865	90.4
Not knoun	4	0.2
**Total**	2,063	100.0

* Increased WC: ≥90 cm for men and ≥80 cm for women.

** Increased WHtR: >0.5.

*** Increased WHR: ≥0.90 for men and ≥0.85 for women.

**** Increased TSF and SSF: >90^th^ percentile of the study population.

Ribeirão Preto, 2002/04.

CS was more common among women with schooling ≥12 (45.1%) when compared with those with 0–4 years (26.8%, p-value<0.0001) or mothers with ≥35 years of age (43.5%) when compared with those <20 (18.4%, p-value<0.0001), and those with birth weight ≥4000 (41.6%) when compared with those <2500 grams (32.2%, p-value = 0.002) (Table 3).

**Table 3:** Type of delivery according to birth variables, 1978/79 Ribeirão Preto birth cohort.

Variables	Vaginal (n = 1,402)*		Cesarean (n = 661)*		P-value**
	n	%	n	%	
Maternal schooling (years)					<0.001
≥12	118	54.9	97	45.1	
9–11	207	62.5	124	37.5	
5–8	373	67.0	184	33.0	
0–4	673	73.2	247	26.8	
Sex					0.985
Male	676	67.9	319	32.1	
Female	726	68.0	342	32.0	
Birth weight (grams)					0.002
<2500	88	68.8	40	32.2	
2500 |–3000	310	74.9	104	25.1	
3000 |–3500	571	67.3	277	32.7	
3500 |–4000	346	66.0	178	34.0	
≥4000	87	58.4	62	41.6	
Maternal smoking during pregnancy (cigarettes/day)					0.762
Non-smoker	1,018	67.5	491	32.5	
1–10	220	69.2	98	30.8	
>10	120	69.4	53	30.6	
Parity					0.300
1	511	66.3	260	33.7	
2–4	739	68.2	344	31.8	
≥5	122	72.2	47	27.8	
Maternal age (years)					<0.001
<20	124	81.6	28	18.4	
20–34	1,169	67.9	553	32.1	
≥35	96	56.5	74	43.5	

* Totals may not add up to 2063 because of missing values.

** P-value refers to the chi-squared test.

CS rate was 32%. Subjects born by CS had greater proportions of increased indicators of adiposity than subjects born by vaginal delivery (p<0.05). Individuals born from mothers with lower schooling levels (0–4 and 5–8 years) also had increased adiposity, except when measured by WHR; men had higher proportions of increased WC, WHtR and WHR, whilst the female gender was associated to increased skinfolds; birth weight ≥4000 g was associated with increased WC but not with other adiposity measures. Multi-parity (≥5) was associated with increased TSF, whilst maternal smoking during pregnancy and maternal age were not associated with increased adiposity (Table 4).

**Table 4:** Distribution of birth variables according to the presence of indicators of increased adiposity in young adults.

Birth variables	*Total*	*Waist Circumference (WC)* *	*Waist-Height Ratio (WHtR)* †	*Waist-Hip Ratio (WHR)* ‡	*Tricipital Skinfold (TSF)* §	*Subscapular Skinfold (SSF)* §
	n (%)	n (%)	n (%)	n (%)	n (%)	n (%)
**Type of delivery**						
Vaginal	1,402 (68.0)	419 (29.9)	434 (31.0)	190 (13.6)	127 (9.1)	118 (8.4)
Cesarean	661 (32.0)	243 (36.8)	247 (37.6)	124 (18.8)	80 (12.1)	76 (11.5)
**P-value**		0.002	0.003	0.002	0.032	0.025
**Maternal schooling (years)**						
≥12	215 (10.6)	55 (25.6)	51 (23.7)	26 (12.1)	12 (5.6)	13 (6.0)
9–11	331 (16.0)	99 (29.9)	105 (31.7)	46 (13.9)	26 (7.8)	23 (7.0)
5–8	557 (27.0)	201 (36.1)	195 (35.1)	93 (16.7)	78 (14.0)	65 (11.7)
0–4	920 (44.6)	296 (32.2)	318 (34.7)	144 (15.7)	89 (9.7)	88 (9.6)
Not known	40 (2.0)					
**P-value**		0.028	0.012	0.363	0.001	0.034
**Sex**						
Male	995 (48.2)	360 (36.2)	407 (41.0)	215 (21.6)	37 (3.7)	76 (7.7)
Female	1,068 (51.8)	302 (28.3)	274 (25.7)	99 (9.3)	170 (15.9)	118 (11.2)
**P-value**		<0.001	<0.001	<0.001	<0.001	0.008
**Birth weight**						
<2500	128 (6.2)	40 (31.2)	46 (35.9)	16 (12.5)	15 (11.7)	12 (9.4)
2500 |–3000	414 (20.1)	114 (27.6)	121 (29.4)	55 (13.3)	38 (9.2)	35 (8.5)
3000 |–3500	848 (41.1)	264 (31.1)	275 (32.5)	129 (15.2)	92 (10.8)	83 (9.8)
3500 |–4000	524 (25.4)	181 (34.5)	177 (33.8)	90 (17.2)	48 (9.2)	48 (9.2)
≥4000	149 (7.2)	63 (42.6)	62 (41.9)	24 (16.2)	14 (9.5)	16 (10.8)
**P-value**		0.011	0.079	0.470	0.762	0.918
Non-smoker	1,509 (73.1)	475 (31.5)	495 (32.9)	226 (15.0)	161 (10.7)	149 (9.9)
1–10	318 (15.4)	113 (35.5)	108 (34.1)	51 (16.1)	23 (7.2)	19 (6.0)
>10	173 (8.4)	57 (33.0)	58 (33.5)	29 (16.8)	20 (11.6)	19 (11.0)
Not known	63 (3.1)					
**P-value**		0.373	0.922	0.762	0.151	0.069
**Parity**						
2–4	1,083 (52.5)	339 (31.3)	353 (32.7)	159 (14.7)	94 (8.7)	94 (8.7)
1	771 (37.4)	255 (33.1)	253 (32.9)	118 (15.3)	89 (11.5)	78 (10.1)
≥5	169 (8.2)	58 (34.3)	63 (37.3)	33 (19.5)	23 (13.6)	18 (10.6)
Not known	40 (1.9)					
**P-value**		0.602	0.497	0.271	0.041	0.487
**Maternal age (years)**						
<20	152 (7.4)	50 (32.9)	57 (37.5)	26 (17.1)	23 (15.1)	17 (11.3)
20–34	1722 (83.5)	545 (31.7)	558 (32.4)	252 (14.6)	161 (9.4)	154 (8.9)
≥35	170 (8.2)	61 (35.9)	63 (37.1)	32 (18.8)	20 (11.8)	19 (11.2)
Not known	19 (0.9)					
**P-value**		0.517	0.234	0.274	0.054	0.427

* Increased WC: ≥90 cm for men and ≥80 cm for women);

† Increased WHtR: >0.5;

‡ Increased WHR: ≥0.90 for men and ≥0.85 for women;

§ Increased TSF and SSF: >90th percentile of the study population.

Ribeirão Preto, 2002/04.

Subjects born by CS had a higher risk for increased adiposity, which persisted even after adjustment for birth variables, with small and non-significant changes. In the adjusted model, babies born by cesarean delivery had an increased risk of 22% for WC, of 25% for WHtR, of 45% for WHR, and of 36% for SSF, and also an increased risk of 43% for peripheral obesity measured by TSF. Models using inverse-probability weighting did not change the estimates appreciably (Table 5). All Poisson models fitted the data well (goodness of fit chi-squared tests were non-significant).

**Table 5:** Association of type of delivery with indicators of increased adiposity in young adults.

*Indicators of increased adiposity*	*Waist Circumference (WC)* *	*Waist-Height Ratio (WHtR)*	*Waist-Hip Ratio (WHR)*	*Tricipital Skinfold (TSF)*	*Subscapular Skinfold (SSF)*
	*IRR (95%CI)* †	*IRR (95%CI)* ‡	*IRR (95%CI)* §	*IRR (95%CI)* #	*IRR (95%CI)* #
Non-adjusted model	1.23 (1.08–1.40)	1.21 (1.07–1.37)	1.39 (1.13–1.71)	1.34 (1.03–1.74)	1.37 (1.04–1.80)
Non-adjusted model using inverse-probability weighting	1.21 (1.06–1.38)	1.20 (1.05–1.36)	1.40 (1.13–1.72)	1.36 (1.04–1.78)	1.41 (1.06–1.87)
Model adjusted for birth variables∞	1.22 (1.07–1.39)	1.25 (1.10–1.42)	1.45 (1.18–1.79)	1.36 (1.04–1.78)	1.43 (1.08–1.91)
Model adjusted for birth variables using inverse-probability weighting∞	1.20 (1.05–1.37)	1.22 (1.08–1.39)	1.42 (1.15–1.76)	1.38 (1.05–1.82)	1.44 (1.08–1.92)

* Increased WC: ≥90 cm for men and ≥80 cm for women);

† RR = Incidence rate ratio; 95%CI = 95% Confidence interval.

‡ Increased WHtR: >0.5;

§ Increased WHR: ≥0.90 for men and ≥0.85 for women;

# Increased TSF and SSF: >90th percentile of the study population;

∞ Birth weight; type of delivery; sex; maternal schooling; maternal smoking during pregnancy; parity; maternal age and gestational age as a continuous variable.

Ribeirão Preto, 2002/04.

## Discussion

The main finding of this study was those individuals born by CS have a higher risk for increased adiposity in adulthood. To our knowledge, this is the first population-based study to show that individuals born by CS had a higher risk for increased central and peripheral adiposity in young adulthood than those born by vaginal delivery. This finding is in accordance with a previous study demonstrating that young adults born by CS had an increased risk of obesity assessed by BMI [7].

Other studies have shown the association between CS and obesity assessed by BMI [6], [7], [8], [9], [10]; however, this is the first study to show the association between CS and other adiposity indicators apart from BMI in young adulthood. Utz et al. (2008) [6] showed that adolescents aged 15 to 19 years born by CS had a 1.4 higher risk of overweight when compared to those born by vaginal delivery. Zhou et al. (2011) [9] found a 5.23 higher risk of obesity in children aged 3 to 6 years born by CS. Rooney et al. [2011] (8) found a 2.5 higher risk at 4–5 years and Huh et al. (2012) (10) found a 2.1 increased risk at age 3. Goldani et al. (2011) [7] found a 1.58 increase risk of obesity in adults aged 23–25 years.

The association of CS and indicators of adiposity in children and adults seems to be consistent both for central markers such as WC, WHtR, WHR and SSF, and for total (BMI) and peripheral (TSF) adiposity; in addition, this association was maintained when adjusted for confounders also considered to be risk factors for adiposity.

However, there are studies in the literature that did not detect an association between CS and obesity in childhood as Ajslev et al. [33]. Barros et al. [34], in a study of three Brazilian cohorts evaluated at the ages of 4, 11, 15 and 23 years, observed an association only for boys at 4 years of age. Rooney et al. [8] observed this association in children but not in adolescents aged 9 to 14 years or in young adults aged 18 to 20 years after adjustment for confounding. On the other side, the meta-analysis by Li et al. (2012) [11], considering such studies and others, taking into account unadjusted and adjusted estimates of the association between CS and overweight and obesity in offspring, indicated CS as a moderate early risk factor for later weight excess (33%) especially in adolescents (24%) and adults (50%). The present study adds this possibility also for central and peripheral adiposity.

The rationale for the association between CS and later obesity is based on the role of gut microbiota. Upon vaginal delivery, the newborn has contact with bacteria from the birth canal first and the infant gut begins to be colonized by an array of bacteria. Infants born by CS lack this contact, which is crucial for the adequate development of the infant's gut microbiota as it is known that its role is related to enhanced availability of nutrients through extraction of calories from luminal oligosaccharides and is also related to improved nutrient uptake by the modulation of absorptive capacity of the intestinal epithelium [35]. Gut microbiota may also promote weight gain and fat accumulation through a condition of low-grade inflammation [18], [19].

Other possible explanation for the association between CS and increased adiposity in adulthood is that absence of the hormonal milieu of labor results in altered metabolic trajectory in the offspring [36].

In a previous analysis of the association between CS and obesity measured by BMI [7] it was stated that a limitation of this study is the lack of data regarding breastfeeding during infancy, as breastfeeding is an important source of bacteria for infant gut development and maturation [37]. CS is seen as a risk factor for early weaning [38], [39], [40], but at the time of the present study, type of delivery was found to have little effect on breastfeeding rates among 6-month-old infants in another Brazilian study [41]. If this were the case in Ribeirão Preto, then the estimates of the present study would have changed little had we included breastfeeding data. Breastfeeding rates are associated with socioeconomic status [42], and the effect of the latter variable on the estimates of adiposity according to type of delivery was controlled for in this study.

The other limitation is the lack of information regarding maternal BMI, which is known to be a risk factor for offspring obesity. This limitation is partially overcome by adjustment for maternal schooling at the time of delivery, because around the time of this study, in 1975, obesity rates in Brazilian women were higher among the wealthy social groups [43]. Alternatively, since CS rate was more incident among mothers of higher schooling, the association between CS and adiposity could be explained by maternal BMI and not by CS, because BMI is an unmeasured confounder. However, this explanation seems unlikely because it is not plausible that BMI could totally explain the association between CS and adiposity.

In a previous analysis it was shown that there was a lower participation of young adults from families with less qualified occupations, of mothers with low schooling levels and smokers during pregnancy [23]. This selective attrition may have biased the association between CS and adiposity. CS was less common in these less privileged population groups [44], maybe leading to a super-estimation of the association. However, adjustment for such variables did not change the associations; in fact, although significant, the differences at birth and in the follow-up were small [23]. Furthermore, CS rates were similar between individuals not included [29.7%] and those included in the analysis at adult age [32%], after exclusion of those who died up to 20 years of age [22]. We implemented the inverse-probability weighting technique that compensates for losses to follow-up. Results did not change appreciably. Thus, it suggests that selective attrition did not bias our conclusions. We also do not have information on CS indication. However, CS rates were higher among the better-off, who are more prone to elective CS – women with higher education levels, who had more prenatal visits, who delivered in private hospitals and during week days [44]. These findings suggest that non-clinical factors were more important in the decision to carry out a CS than medical factors, as stated by others around the time of this study [45]. Since we do not have data on CS with and without labor, we were not able to test if the association between CS and increased adiposity in adulthood is driven by emergency or elective CS, or both.

The strengths of the present study are as follows: the narrow age group (from 23 to 25 years of age) is a particular strength because it eliminates the confounding effect of age and many other age-dependent covariates that may have affected the analysis. Confounding variables from birth were adjusted for, particularly those related to socioeconomic status. This adjustment may have possibly minimized the effect of unmeasured confounding factors such as early weaning and maternal obesity, both more prevalent in the better off groups of women at this time.

In conclusion, subjects born by CS had a higher risk for increased central and peripheral adiposity in young adulthood than those born by vaginal delivery. The association of CS with adiposity was consistently observed for all indicators and was robust to adjustment for a variety of early life confounders. Results did not change appreciably after inverse-probability weighting, indicating that selective attrition did not bias our conclusion. Possible mechanisms to explain this association are changes in gut microbiota induced by CS or lack of the hormonal milieu during labor, which may increase the risk of obesity in later life.
